# Purification of a Lectin from *Arisaema erubescens* (Wall.) Schott and Its Pro-Inflammatory Effects

**DOI:** 10.3390/molecules16119480

**Published:** 2011-11-14

**Authors:** Xian Qiong Liu, Hao Wu, Hong Li Yu, Teng Fei Zhao, Yao Zong Pan, Run Jun Shi

**Affiliations:** 1 Jiangsu Key Laboratory of Chinese Medicine Processing, Nanjing University of Chinese Medicine, Nanjing, 210046, China; 2 Hubei University of Chinese Medicine, Wuhan, 430061, China

**Keywords:** *Arisaema erubescens* lectin (AEL), haemagglutination activity, paw edema, neutrophil migration, pro-inflammatory compounds

## Abstract

The monocot lectin from the tubers of *Arisaema erubescens* (Wall.) Schott has been purified by consecutive hydrophobic chromatography and ion exchange chromatography methods. The molecular weight of this *A. erubescens* lectin (AEL) was determined to be about 12 kDa by high performance liquid chromatography (HPLC) and sodium dodecyl sulphate polyacrylamide gel electrophoresis (SDS-PAGE) methods. AEL could agglutinate rabbit erythrocytes. The haemagglutination activity of AEL was only inhibited by asialofetuin, while monosaccharide did not react. Rat paw edema and neutrophil migration models were used to investigate the pro-inflammatory activity of AEL. AEL (100 and 200 μg/paw) could induce significant rat paw edema. In addition, AEL (100, 200 and 300 μg/mL/cavity) could induce significant and dose-dependent neutrophil migration in the rat peritoneal cavities. Besides, AEL at doses ranging from 100 to 300 μg/mL/cavity could significantly increase the concentration of nitric oxide (NO), prostaglandin E_2_ (PGE_2_) and tumor necrosis factor alpha (TNF-α) in peritoneal fluid. As compared with control animals, 75% depletion in the number of resident cells following peritoneal lavage did not reduce the AEL-induced neutrophil migration. However, pre-treatment with 3% thioglycollate which increased the peritoneal macrophage population by 201%, enhanced the neutrophil migration induced by AEL (200 μg/mL/cavity) (*p* < 0.05). Reduction of peritoneal mast cell population by chronic treatment of rat peritoneal cavities with compound 48/80 (*N*-methyl-*p*-methoxyphenethylamine with formaldehyde) did not modify AEL-induced neutrophil migration. The results provided the basis for identifying the toxic components of *A. erubescens* and AEL could be a new useful tool for pro-inflammatory research.

## 1. Introduction

Arisaematis Rhizoma (AR) is the rhizomes of *Arisaema erubescens* (Wall.) Schott, which has been widely used in Traditional Chinese Medicine for thousands of years. Chemical research showed that AR contained alkaloids, saponins, triterpenoids [1] and lectins [2]. AR exhibited the abilities of eliminating dampness, resolving phlegm, expelling wind, relieving convulsions, removing swelling and lumps. However, it has been reported that AR possesses toxic properties, such as causing mucous membrane and skin irritation, mouth and lingua pain, even respiration slowing and suffocation, which have seriously restricted the development of its clinical applications. Our previous research had proved that AR demonstrated toxicity due to the components of raphide including calcium oxalate, protein and trace carbohydrates [3,4]. Moreover, investigations showed that the toxicity of the raphide might closely relate to the protein components [5]. Among the protein components, a lectin was proven to be the main pro-inflammatory component [6]. Lectins are (glyco) proteins of non-immune origin that interact reversibly and specifically with carbohydrates. Lectins have various biological activities, such as anticancer [7], immunomodulatory [8], antifungal [9], antiviral [10] and anti-insect activity [11]. Moreover, lectins have been shown to present stimulatory effects in different biological models. Lectin from *Vatairea macrocarpa* could induce paw edema in rats [12]. Furthermore, it could induce neutrophil migration *in vivo* [13]. Similar effects have also been observed for the plant lectins from *Arum maculatum* [14] and *Pisum arvense* [15]. However, there are few reports on the toxic components and its toxicity mechanisms of AR. Therefore, it was deemed necessary to investigate the toxic components of AR in order to ensure a more safe and effective use in clinical treatment.

In this study, lectin from the roots of *A. erubescens* (AEL) was purified and its haemagglutination activity was also investigated. The pro-inflammatory effects of AEL were evaluated by rat paw edema and neutrophil migration into rat peritoneal cavity models*.* In addition, the contents of inflammatory mediators such as nitric oxide (NO), prostaglandin E_2_ (PGE_2_) and tumor necrosis factor alpha (TNF-α) have also been determined. Finally, the relationship of AEL-induced neutrophil migration and the possible involvement of resident cells, macrophages as well as mast cells were investigated. 

## 2. Results and Discussion

### 2.1. Extraction and Purification of AEL

**Figure 1:**
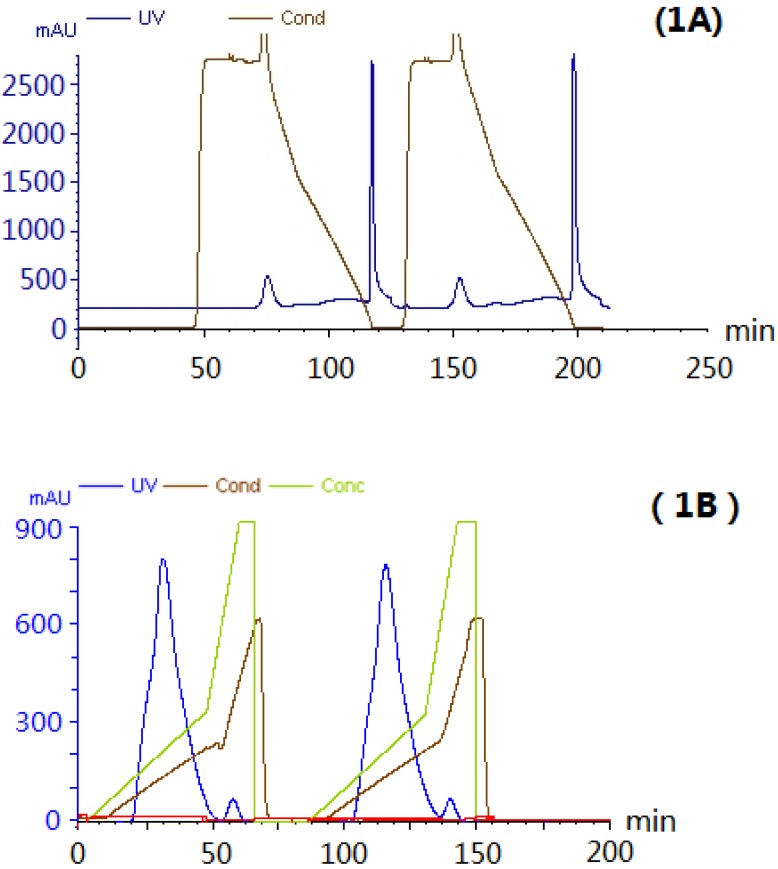
The purification of AEL by hydrophobic interaction chromatography and ion exchange chromatography. The elution profiles were monitored at 280 nm. (**A**) Hydrophobic interaction chromatography of protein on Hiprep^TM^ Phenyl FF column (10 mL). The bound protein was eluted with a linear gradient of 0.6–0.3 mol/L (NH_4_)_2_SO_4_ for 15 min, then with 0.3–0 mol/L (NH_4_)_2_SO_4_ for 30 min, finally with H_2_O at a flow rate of 1 mL/min; (**B**) Ion exchange chromatography on HiTrap^TM^ column pre-equilibrated with Tris-HCl buffer [pH 8.0]. The main peak which was obtained by hydrophobic interaction chromatography was eluted with a linear gradient of 0–0.4mol/L NaCl at a flow rate of 1 mL/min.

**Figure 2:**
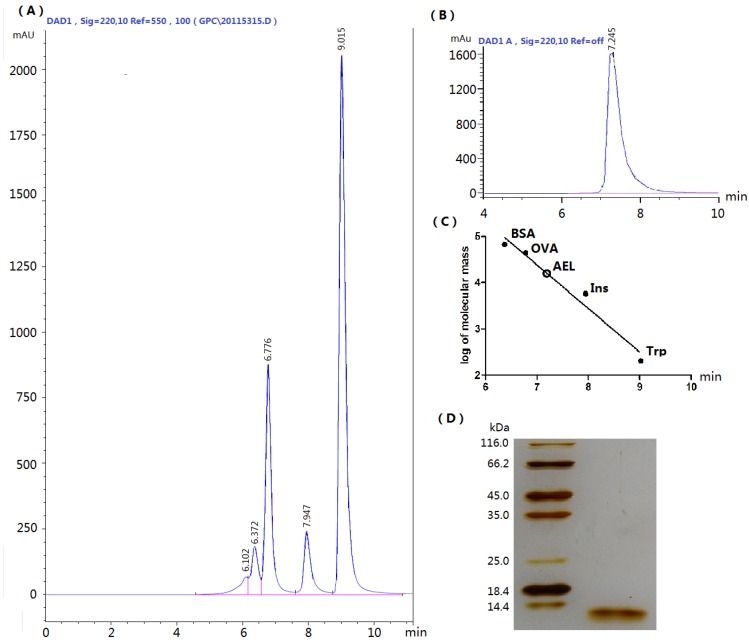
(**A**) Standard proteins analyzed by size-exclusion HPLC chromatography. Standard markers: bovine serum albumin (BSA, 67 kDa), ovalbumin (OVA, 44 kDa), insulin (Ins, 5.7 kDa) and tryptophan (Trp, 0.2 kDa); (**B**) AEL by size-exclusion HPLC; (**C**) Standard curve representing native molecular weight of AEL by size-exclusion HPLC; (**D**) SDS-PAGE, pH 8.3 purified AEL using 12% gel (running time 3 h at a constant 100 V). Mr, Molecular weight markers (from top to bottom): β-galactosidase (116.0 kDa); bovine serum albumin (66.2 kDa); ovalbumin (45.0 kDa); lactate dehydrogenase (35.0 kDa); REase Bsp981 (25.0 kDa); β-lactoglobulin (18.4 kDa); and lysozyme (14.4 kDa). The gels were stained with silver nitrate.

Hydrophobic interaction, ion exchange and desalting chromatographic steps were applied to the purification of AEL. The crude protein extract was eluted by hydrophobic interaction. The main peak (Figure 1A) was eluted with a linear gradient of NaCl (0–0.4 mol/L) (Figure 1B). A single peak on HPLC and a single band of about 12 kDa on SDS-PAGE (Figure 2) were observed, suggesting that the purity of isolated AEL was fairly good.

The purification method was suitable, which indicated that AEL contained hydrophobic amino acid residues like the *Arisaema lobatum* lectin [16]. To study the purification factor, the buffer system was checked by testing the resolution using ion exchange chromatography, such as Tris-HCl [pH 8.0], PBS [pH 7.2] and Bis-Tris [pH 6.0]. AEL was active at Tris-HCl [pH 8.0] buffers. This result indicated that this special chromatographic purification procedure could be suitable for AEL.

### 2.2. Haemagglutination Assays

AEL agglutinated rabbit erythrocytes. The haemagglutination activity of AEL was only inhibited by asialofetuin, while monosaccharide did not react. The minimal inhibitory concentration of asialofetuin for AEL (50 μg/mL) was 1 mg/mL. Interestingly, although asialofetuin binded to the lectin, the monosaccharides constituting the oligosaccharide chains of asialofetuin, such as galactose and mannose, still failed to recognize AEL, even when tested at a final concentration of 2 mol/L. Inhibition of haemagglutination with asialofetuin but not fetuin might suggest that sialic acid hinders the binding of AEL to the recognition sites on fetuin. Just like the monocot lectins from *Arisaema consanguineum* Schott, *Arisaema curvature* Kunth, *Sauromatum guttatum* Schott and *Gonatanthus pumilus* D. Don [2] whose inhibition by asialofetuin only were established, although another lectin from *Clematis montana* [17] has been reported to have haemagglutinating activity inhibited by yeast mannan and man-α(1,3:1,6)-mannotriose.

The haemagglutinating activity of AEL was stable up to 70 °C for 20 min without any loss of activity, but it decreased sharply between 80 °C and 90 °C and was completely abolished when incubated at 100 °C for less than 10 min. Agglutination was not markedly affected by pH in the range of 6.0–10.0 (data not shown). Although AEL constituted only a small proportion of the total weight of tubers, it represented a considerable proportion of the tuber protein suggesting that such a high lectin content may fulfill some physiological role in the plant.

### 2.3. AEL-Induced Rat Paw Edema

**Figure 3:**
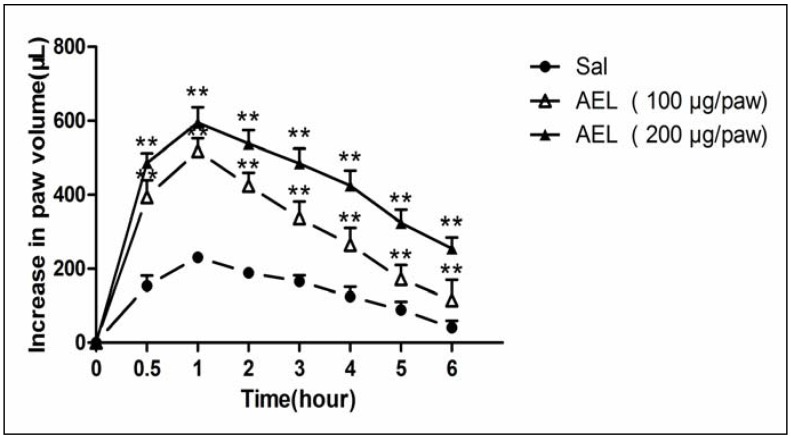
AEL induces rat paw edema. AEL(100 and 200 μg/paw) was injected in the animal right hind paws (s.c. intraplantar). Edema was measured 0.5, 1, 2, 3, 4, 5 and 6 h after AEL injection and expressed as the mean ± S.D. (n = 6) of the increase in paw volume (mL). ** *p* < 0.01 compared with animals injected with saline only (Sal).

AEL caused a pronounced edema with a peak at 30 min after injection and reached a maximum at about 1 h, followed by a decrease (Figure 3). AEL was efficient at the doses of 200 μg/paw, evoking an increase in animal paw volumes of 152%, compared with the control group. However, AEL-induced edematogenic effect was still significant even 6 h after injection. Lectins from *Dioclea grandiflora* and *Canavalia brasiliensis* were effective in inducing rat paw edema and the effect was still significant even after 48 h [18]. It has been demonstrated that leukocyte recruitment is potentially able to contribute to the development of the edema by releasing several mediators of an acute inflammatory response [12].

### 2.4. AEL-Induced Neutrophil Migration and NO, PGE_2_, TNF-α in Vivo

**Figure 4:**
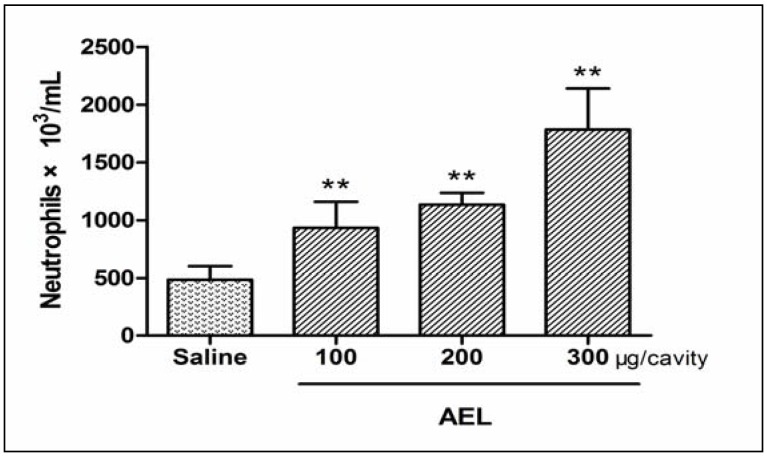
Dose dependent neutrophil migration induced by AEL. AEL (100, 200 and 300 μg/cavity in 1 mL of saline), or saline (1 mL/cavity) were injected into peritoneal cavities. After 4 h, the exudates were collected by washing with 10 mL of saline containing 8 UI/mL heparin. Total and differential cells counts were performed. The results are mean ± S.D. (n = 6). ** *p* < 0.01 compared with saline.

**Figure 5:**
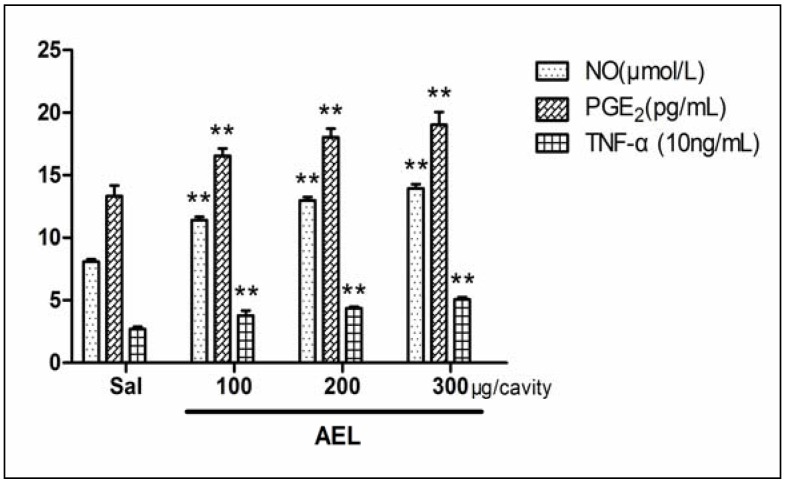
Neutrophil chemotaxis induced by AEL (100, 200 and 300 μg/mL) was assayed. NO was evaluated by nitrite concentrations using the Griess reaction, PGE_2_ and TNF-α were measured by radioimmunoassay. Results are reported as the mean ± S.D. (n = 6) ** *p* < 0.01 compared with saline.

The intraperitoneal (i.p.) injection of AEL (100, 200 and 300 μg/cavity in 1 mL of saline) in rats caused signiﬁcant and dose-dependent neutrophil migration 4 h after injection as compared with saline group (Figure 4). The range of doses chosen was based on previous publication using the lectin from *Pisum arvense* seeds [15]. In order to examine which inflammatory mediators may be involved in AEL-induced neutrophil migration, the effects of several different inflammatory mediators such as NO, PGE_2_ and TNF-α were tested (Figure 5). It has been described that plant lectins are able to release NO production *in vivo* and *in vitro* [19]. NO, PGE_2_ and TNF-α have emerged as the major effect molecules of murine macrophage cytotoxicity [20] and the cytotoxic activity of induced macrophages can be characterized by measuring NO, PGE_2_ and TNF-α release [21,22,23].

### 2.5. AEL-Induced Neutrophil Migration with Resident Cells

**Figure 6:**
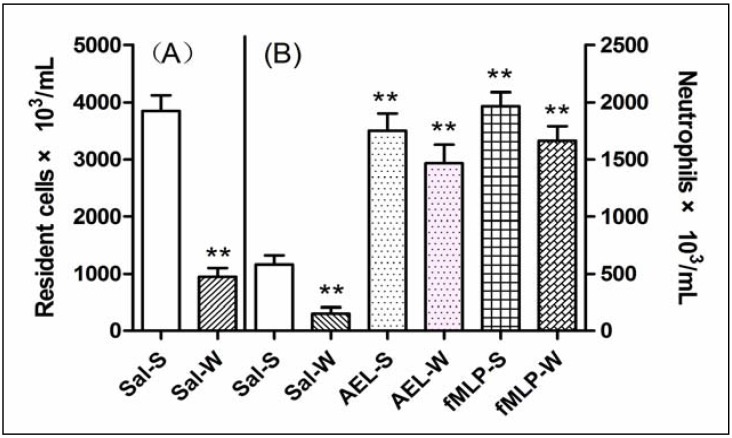
Reduction of resident cell population by peritoneal wash inhibits the neutrophil migration induced by AEL. (**A**) Number of mononuclear cells in sham (S) and washed (W) peritoneal cavities; (**B**) Neutrophil migration induced by saline (Sal), AEL (200 μg/cavity in 1 mL of saline) and fMLP (10^−7^ mol) into sham (S) and washed (W) cavities. The results are reported as mean ± S.D. (n = 6). ** *p* < 0.01 compared with saline.

**Figure 7:**
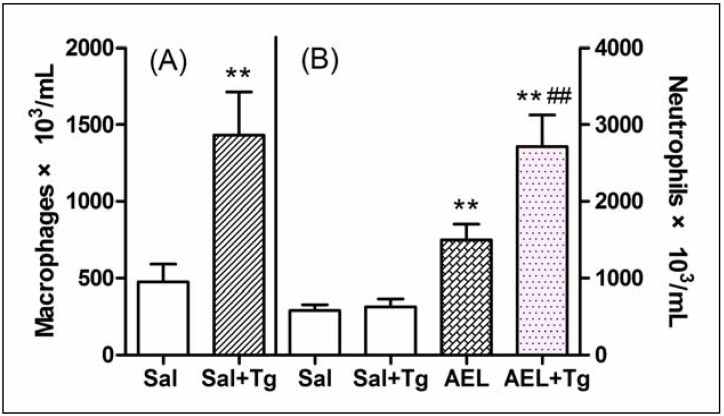
Thioglycollate potentiates the neutrophil migration induced by AEL. (**A**) Macrophage population in normal (Sal) and in Tg-pretreated animals (Sal + Tg); (**B**) Neutrophil migration induced by saline (1 mL/cavity) and AEL(200 μg/cavity in 1 mL saline) in normal (Sal and AEL) and in Tg-pretreated animals (Sal + Tg and AEL + Tg). Results are mean ± S.D. (n = 6). ** *p* < 0.01 compared with saline and ^##^ *p* < 0.01 compared with AEL.

**Figure 8:**
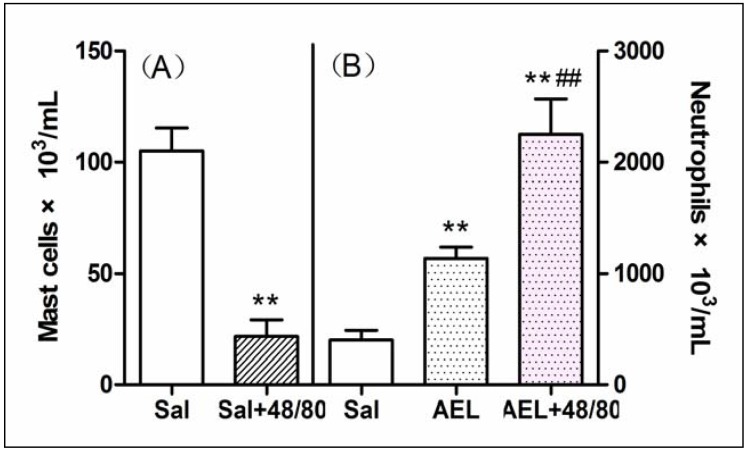
Analysis of the participation of mast cells on the neutrophil migration induced by AEL (200 μg/mL/cavity). (**A**) Peritoneal mast cell population in normal (Sal) or pre-treated animals with compound 48/80 (Sal + 48/80) after injection with 1 mL saline; (**B**) Neutrophil migration induced by 1mL of saline (Sal) and AEL (200 μg/cavity in 1 mL of saline) in normal (AEL) or compound 48/80 pre-treated animals (AEL + 48/80). Results are mean ± S.D. (n = 6). ** *P* < 0.01 compared with saline and ^##^ *P* < 0.01 compared with AEL.

The role of resident cells on the AEL effect was evaluated using three strategies: (a) reducing the total resident cell population by lavage of rat cavities with saline; (b) increasing macrophage population by treating rats with Tg; (c) depleting mast cell population by subchronic treatment of rats with compound 48/80. As compared with sham animals (not washed), depletion of 75% of total resident peritoneal cells by previous lavage of the cavities with saline did not decrease the neutrophil migration induced by AEL (200 μg/mL/cavity), similar to that produced by fMLP (10^−7^ mol), a classical direct neutrophil chemoattractant (Figure 6). Surprisingly, when the peritoneal macrophage population was increased by pre-treatment of the animals with Tg, AEL induced neutrophil migration was significantly increased (Figure 7). This result might indicate that AEL seems to stimulate macrophages to release neutrophil chemotactic factors. Moreover, the migration of neutrophil cells induced by AEL into the peritoneal cavities increased when mast cell population was reduced by pre-treatment of rats with compound 48/80 (Figure 8). It is possible that AEL does not directly activate mast cells, but could be releasing inhibitory neutrophil chemotactic factors. These findings suggested that AEL-induced neutrophil migration follows an indirect pathway and may be dependent on the release of neutrophil chemotactic factors from resident macrophages. It is believed that in early stages of the inflammatory process, induced by different stimuli, tissue resident cells such as macrophages, mast cells and lymphocytes participate in the control of neutrophil migration.

The interactions among macrophages, mast cells and neutrophils under inflammatory stimulus induce the release of inflammation mediators which are responsible for the development and control of the inflammatory reaction [24,25,26]. It has been described that plant lectins are able to release neutrophil chemoattractant factors dependent on macrophage activation [13,14]. 

In this paper, it is reasonable to suggest that AEL can induce rat paw edema and neutrophil migration, probably via the release of neutrophil chemotactic factors raised from resident macrophages. These data also provide additional evidence to prove that AEL is able to induce NO, PGE_2_ and TNF-α synthesis *in vivo*. This control is mediated via release of chemotactic factors such as leukotrienes [26], components of the complement system [27]. In inflammatory reactions induced by exogenous stimuli such as carrageenan, zymosan and lipopolysaccharide (LPS) or by chemotactic mediators such as IL-1 or TNF-α, which induce neutrophil migration by indirect mechanisms, resident macrophages are believed to be required for the control of neutrophil recruitment [28,29]. Since AEL-induced neutrophil migration seems to follow an indirect pathway, mediated by macrophages, the AEL inducing effects may be explained by the release of inflammation mediators, stimulated by inflammatory stimuli [25], and AEL thus possesses pro-inflammatory activity. Similar effects have also been observed in the plant lectins from *Arum maculatum* [14] and *Pisum arvense* [15], although another lectin from *Luetzelburgia auriculata* could inhibit paw edema in rats [30]. Perhaps different administration routes, different sugar residues combining with the lectins and different inflammatory inductors [31] could influence the effects of lectins. It is postulated that these findings favour the hypothesis that AEL-induced neutrophil migration may possibly be mediated via release of inflammatory mediators by resident macrophages. Damage to the three-dimensional structure of the lectin after exposure to heat or alkali removed its biological activities, including haemagglutinating activity. These findings may explain why heat and lime water [pH 12] pre-treatment are necessary for the clinical use of AR.

## 3. Experimental Section

### 3.1. Plant Material

Fresh tubers of *A. erubescens* were collected from Yuanling County, Hunan Province, China and identified by Chungen Wang (Nanjing University of Chinese Medicine, China) and stored at −40 °C until use.

### 3.2. Animals

Male Sprague-Dawley rats and rabbits bought from the Experimental Animal Center of Nanjing University of Chinese Medicine (Jiangsu, China). All animals used in these studies were kept in an environmentally controlled breeding room (temperature maintained at about 25 °C and with a 12 h light/12 h dark cycle) for at least one week before the experiments and fed with standard laboratory food and water *ad libitum*. Prior to each experiment, the rats were fasted for 12 h with free access to water. Animal welfare and experimental procedures were strictly in accordance with the Guide for the Care and Use of Laboratory Animals (US National Research Council, 1996) and the related ethics regulations of Nanjing University of Chinese Medicine.

### 3.3. Apparatus, Chemicals and Reagents

Mini protean cell was supplied by Bio-Rad (Hercules, California, USA) Hiprep^TM^ Phenyl FF, HiTrap^TM^ Q FF, HiTrap^TM^ Desalting and AKTA purifier were supplied by GE (Uppsala, Sweden). Tris(hydroxymethyl) aminomethane, asialofetuin from fetal calf serum, methyl-α-d(+)-mannopyranoside, fluid thioglycolate (Tg) medium, compound 48/80 (*N*-methyl-*p*-methoxyphenethylamine with formaldehyde), and *N*-formylmethionyl-leucyl-phenylalanine (fMLP) were obtained from Sigma (St. Louis, MO, USA). Distilled water was produced in EPED Superpure water purifying system (Nanjing, China). Sodium chloride, ammonuium sulfate and sodium hydroxide were of analytical grade and obtained from Nanjing Chemical Reagent Co., Ltd (Jiangsu, China). All the reagents were at least analytical grade.

### 3.4. Extraction and Purification of AEL

Fresh tubers of *A. erubescens* weighing 100 g were washed thoroughly with tap water and then with distilled water. The combined roots and tubers were separated from the stems and leaves before they were crushed using a juice extractor to extract the juice and then centrifuged for 20 min at 4 °C. The clear supernatant was dissolved with saturated (NH_4_)_2_SO_4_ [pH 7.0] and then centrifuged for 30 min at 4 °C. The precipitate was full dissolved with 0.6 mol/L (NH_4_)_2_SO_4_ [pH 7.0] and centrifuged at high speed. The resulting supernatant obtained was applied to a Hiprep^TM^ Phenyl FF (10 mL) column. The mobile phase was 0.6–0 mol/L (NH_4_)_2_SO_4_ gradient at a ﬂow rate of 1.0 mL/min. Then main peak was collected and applied to a column of HiTrap^TM^ Q FF. The column was eluted with gradient of 0–0.4 mol/L NaCl at a flow rate of 1.0 mL/min. Finally main peak was desalted with 0.02 mol/L Tris-HCl [pH 8.0] buffer for lectin purification and subsequently freeze-dried.

### 3.5. SDS-PAGE and SEC-HPLC

Purified lectin preparation was subjected to SDS-PAGE [pH 8.3], using 12% (w/v) acrylamide slab gel for subunit molecular mass determination as described by Laemmli [2]. The sample was heated for 5 min in a boiling water bath. The gel was stained with silver nitrate. Destained gel was scanned using a Samsung camera (Seoul, Korea). The molecular weights of the standard marker proteins were matched with the sample protein (14.4–116.0 kDa) to determine the subunit molecular weight of AEL. AEL was also checked on an Agilent 1200 system equipped with DAD detector using an Agilent Zorbax GF-450 column (9.4 × 250 mm). The mobile phase was 0.1 mol/L phosphates buffer at a ﬂow rate of 1.5 mL/min.

### 3.6. Haemagglutination and Inhibition Assays

Haemagglutinating activity of AEL was assayed in 96-well microtiter plates according to the serial double dilution method using 2% suspension of rabbit erythrocytes [32]. AEL (40 μL) was serially diluted 2-fold in 0.15 mol/L NaCl and an equal volume of erythrocytes in suspension was added to the microtiter plates. The mixture was incubated for 1 h at room temperature before the plate was read. The haemagglutination titer was defined as the reciprocal of the highest dilution exhibiting haemagglutination. Haemagglutination inhibition assays were analyzed in a manner analogous to the haemagglutination assays. Briefly, serial 2-fold dilutions of sugar samples at doses ranging from 0.2 mol/L to 2 mol/L such as methyl-α-d(+)-mannopyranoside, D-xylose, D-ribose, D-arabinose, D-glucose anhydrous, D-galactose, D-mannose, L-rhapontin and asialofetuin at doses ranging from 1 mg/mL to 10 mg/mL were mixed with an equal volume of the lectin in microtiter plates and incubated for 30 min at 37 °C. Then 40 μL erythrocytes suspension was added to each well, mixed and the plates read after 1 h. The minimum concentration of the sugar in the final reaction mixture for complete inhibition of the lectin preparation was calculated [33].

### 3.7. Thermal Stability

The purified lectin was incubated in water bath at 20 °C for 15 min, with 5 °C increase at each step up to 100 °C. After each step of incubation, a haemagglutination assay was done to study the effect of temperature.

### 3.8. pH Stability

The pH stability of AEL was determined by extensive dialysis of AEL (1 mg/mL) against buffers of different pH values ranging from pH 4.5–11.0. The pH of AEL solution was adjusted to pH 7.0 by the addition of 0.1 mol/L HCl or 0.1 mol/L NaOH before haemagglutination activity was determined [7]. 

### 3.9. Rat Paw Edema

Paw edema was induced by sub plantar injection of AEL (100, 200 μg/paw), dissolved in sterile 0.15 mol/L NaCl (saline), at a final volume of 0.1 mL into the right hind paw of rats under light ether anesthesia. Edema was measured plethysmographically according to Ferreira [34]. Paw volume was measured immediately before AEL injections and at selected time intervals thereafter (0.5, 1, 2, 3, 4, 5 and 6 h) with a hydroplethysmometer. Results were expressed as the increase in paw volume (μL) calculated by subtracting the basal volume. 

### 3.10. Stimulation of Neutrophil Migration into Peritoneal Cavities by AEL

AEL was injected intraperitoneally (i.p.) at 100, 200 or 300 μg/cavity in 1 mL of 0.15 mol/L NaCl (saline) in three group of rats. In the control animals the same volume of saline solution, which contained no protein, was injected. Four hours later, animals were sacrificed and peritoneal cells harvested by washing each peritoneal cavity with 10 mL of saline containing 8 UI/mL heparin [28]. Total and differential cell counts were performed as described using hematology system with Bayer 120. 

### 3.11. Content of NO, PGE_2_, TNF-α Induced by AEL

The amount of NO, PGE_2_ and TNF-*α* in the peritoneal cavities previously described in *3.10* were further measured. The presence of NO in supernatants of rat peritoneal cavities was evaluated by nitrite concentrations using the Griess reaction (Nanjing Jiancheng Bioengingeering Institute). Optical density readings at 550 nm were performed using a microplate reader. PGE_2_ and TNF-*α* were determined in supernatants of rat peritoneal cavities by radio immunoassay (Beijing Sino-UK Institute of Biological Technology).

### 3.12. Depletion of Total Resident Cell Population by Peritoneal Lavage

The number of resident cells was diminished by lavage with sterile saline as described in the classical method [35]. Rats were anaesthetized with ethyl ether and three hypodermic needles were inserted into their abdominal cavities. Thirty mL of sterile saline were injected through the needle placed nearest the sternum. The abdominal cavity was then gently massaged for 1 min and the peritoneal fluid was collected via the two needles inserted into the inguinal region. This operation was repeated three times. Control (sham; S) rats were impaled and manipulated in the same way but no fluid was injected or withdrawn. After 30 min, resident cells were estimated by injecting 10 mL of saline containing 8 UI/mL heparin [25]. Both sham and washed animals received saline (1 mL/cavity), AEL (200 μg/cavity in 1 mL of saline) or fMLP (10^−7^ mol) and the neutrophil migration was estimated 4 h later.

### 3.13. Increase of the Peritoneal Macrophage Population by Treatment with Tg

Tg (3%, w/v; 1 mL i.p.) was injected into the peritoneal cavities and after four days peritoneal macrophages were collected, counted and compared to those from a group of non-treated animals (control) [24]. Saline (1 mL/cavity) or AEL (200 μg/cavity in 1 mL of saline), was then injected into rats (control and Tg treated), and 4 h later, the neutrophil migration was evaluated. The number of neutrophils in the peritoneal washes collected from control rats, four days after Tg treatment were subtracted from the number of neutrophils counted after administration of AEL to Tg-treated animals.

### 3.14. Depletion of Peritoneal Mast Cell Population by Chronic Treatment with Compound 48/80

Peritoneal mast cell population was depleted by chronic treatment with compound 48/80 according to the method of Di Rosa, Giround, and Willoughby [36]. For this, animals were treated with compound 48/80 during four days (0.6 mg/kg, i.p., twice a day for three days and 1.2 mg/kg, i.p., twice a day on the 4th day). At the 5th day, the depletion of mast cell population in a selected group of animals was assessed by conventional light microscopy after staining the cells with toluidine blue. Final counts were compared to those obtained from a group of non-treated rats (control). Saline (1 mL/cavity) or AEL (200 μg/cavity in 1 mL of saline), was then injected into both control and compound 48/80 treated animals and after 4 h the neutrophil migration induced by these chemotactic stimuli was evaluated.

### 3.15. Statistical Analysis

Results are expressed as mean ± S.D. Data were tested for normal distribution and analyzed by Student’s t-test. All experiments were performed at least three times and one representative experiment is presented. All *p*-values were two-tailed and *p*-values less than 0.05 presented statistical significance.

## 4. Conclusions

The results reported here clearly demonstrate that the chosen purification method was suitable for separating AEL. AEL possesses pro-inflammatory activity, which induces rat paw edema and neutrophil migration, probably via the release of inflammatory mediators from macrophages. These findings indicate AEL could be used as a tool for better understanding of the mechanisms involved in the inflammatory response.
